# Bioinformatics approaches and applications in plant biotechnology

**DOI:** 10.1186/s43141-022-00394-5

**Published:** 2022-07-15

**Authors:** Yung Cheng Tan, Asqwin Uthaya Kumar, Ying Pei Wong, Anna Pick Kiong Ling

**Affiliations:** 1 Division of Applied Biomedical Sciences and Biotechnology, School of Health Sciences, International Medical University, 126 Jalan Jalil Perkasa 19, Bukit Jalil, 57000 Kuala Lumpur, Malaysia; 2 School of Biosciences and Biotechnology, Faculty of Science and Technology, Universiti Kebangsaan Malaysia, 43600 Bangi, Malaysia

**Keywords:** Bioinformatics, Biotic and abiotic, GWAS, NGS, Plant breeding, Plant sequencing, Plant pathogen, PRGdb sequence analysis

## Abstract

**Background:**

In recent years, major advance in molecular biology and genomic technologies have led to an exponential growth in biological information. As the deluge of genomic information, there is a parallel growth in the demands of tools in the storage and management of data, and the development of software for analysis, visualization, modelling, and prediction of large data set.

**Main body:**

Particularly in plant biotechnology, the amount of information has multiplied exponentially with a large number of databases available from many individual plant species. Efficient bioinformatics tools and methodologies are also developed to allow rapid genome sequence and the study of plant genome in the ‘omics’ approach. This review focuses on the various bioinformatic applications in plant biotechnology, and their advantages in improving the outcome in agriculture. The challenges or limitations faced in plant biotechnology in the aspect of bioinformatics approach that explained the low progression in plant genomics than in animal genomics are also reviewed and assessed.

**Conclusion:**

There is a critical need for effective bioinformatic tools, which are able to provide longer reads with unbiased coverage in order to overcome the complexity of the plant’s genome. The advancement in bioinformatics is not only beneficial to the field of plant biotechnology and agriculture sectors, but will also contribute enormously to the future of humanity.

## Background

Over the past decades, the term ‘bioinformatics’ has become a buzzword in all areas of research in biological science. With the continuous development and advancement in molecular biology, the explosive growth of biological information required a more organized, computerized system to collect, store, manage, and analyse the vast amount of biological data generated in the experiments from all fields [1]. Bioinformatics, as a new emerging interdisciplinary field for the past few decades, has many tools and techniques that are essential for efficient sorting and organizing of biological data into databases [1, 2]. Bioinformatics can be referred as a computer-based scientific field which applies mathematics, biology, and computer science to form into a single discipline for the analyses and interpretation of genomics and proteomics data [2, 3]. In short, the main components of bioinformatics are (a) the collection and analysis of database and (b) the development of software tools and algorithm as a tool for interpretation of biological data [2]. Bioinformatics played a crucial role in many areas of biology as its applications provide various types of data, including nucleotide and amino acid sequences, protein domains and structure as well as expression patterns from various organisms [3]. Similarly, the field of plant biotechnology has also taken advantages of bioinformatics, which provides full genomic information of various plant species to allow for efficient exploration into plants as biological resource to humans [1, 3, 4]. The intention of this article is to describe some of the key concepts, tools, and its applications in bioinformatics that are relevant to plant biotechnologies. The current challenges and limitations for improvement and continuous development of bioinformatics in plant science are also described.

## Main text

### Applications of bioinformatics in plant biotechnology

The introduction of bioinformatics and computational biology into the area of plant biology is drastically accelerating scientific invention in life science. With the aid of sequencing technology, scientists in plant biology have revealed the genetic architecture of various plant and microorganism species, such as proteome, transcriptome, metabolome, and even their metabolic pathway [1]. Sequence analysis is the most fundamental approach to obtain the whole genome sequence such as DNA, RNA, and protein sequence from an organism’s genome in modern science. The sequencing of whole genome permits the determination of organization of different species and provides a starting point to understand their functionality. A complete sequence data consists of coding and non-coding regions, which can act as a necessary precursor for any functional gene that determines the unique traits possessed by organisms. The resulting sequence includes all regions such as exons, introns, regulator, and promoter, which often leads to a vastly large amount of genome information [5]. With the emergence of next-generation sequencing (NGS) and some other omics technologies used to examine plants genomics, more and more sequenced plants genome will be revealed [1, 6–8]. To deal with these vast amounts of data, the development and implementation of bioinformatics allow scientists to capture, store, and organize them in a systematic database [1, 5].

### Bioinformatics databases and tools for plant biotechnology

In the field of bioinformatics, there are a variety of options of databases and tools that are available to perform analysis related to plant biotechnology. Next-generation sequencing (NGS) and bioinformatics analysis on the plant genomes over the years have generated a large amount of data. All these data are submitted to various and multiple databases that are publicly available online. Each database is unique and has its focus. For instance, CottonGen, database is solely dedicated to obtaining genomics and breeding information of any cotton species of interest [9]. The establishment of such database eases the researchers who are working on cotton genomic studies by focussing on using just one database instead of searching through other available databases. However, some databases are established and designed to cater not only to one specific species or genus, but focus on all the plant species, such as the National Center for Biotechnology Information (NCBI) (https://www.ncbi.nlm.nih.gov/) database, which as of 2021 possesses almost 21,000 plant genomes that are available for access [10]. Such a database is useful for studies that do not focus on one specific genus or species. This eases the researchers in accessing to all kinds of genomic data in one database. This section will briefly discuss some of the available plant genome databases, which are publicly accessible and not designated for one genus or species alone.

First would be the globally known and recognized database by all the researchers and biologists, which is the NCBI database. NCBI has been dedicated for gathering and analysing information about molecular biology, biochemistry, and genetics. In the NCBI database, one can download the genome information of the plant species of interest from either gene expression omnibus (GEO) (https://www.ncbi.nlm.nih.gov/geo/) or sequence read archive (SRA) (https://www.ncbi.nlm.nih.gov/sra) by simply stating the scientific name of the plant in the search bar and the entire genomic information of the plant can then be obtained. The GEO and SRA comprise processed or raw gene expression data or RNA sequencing of plants that are reposited in the repository. For instance, to obtain the genomics of *Rosa chinensis* (Rose plant), by inputting the name in the search bar, it will direct to the search result page where the researcher can select the most recent or suitable datasets with specific accession number. Depending on the profiling platform used in each dataset, researchers could retrieve either gene symbols, Ensemble ID, open reading frame, chromosomal location, regulatory elements, etc. The information allows researcher to further analyse the subject of study using bioinformatics tools such as gene ontology (http://geneontology.org/), Database for Annotation, Visualization and integration Discovery (DAVID) (https://david.ncifcrf.gov/), Basic Local Alignment Search Tool (BLAST) (https://blast.ncbi.nlm.nih.gov/Blast.cgi), and others that is relevant for the study.

Another database that is available for accessing plant genome database is EnsemblPlants (https://plants.ensembl.org/index.html). Unlike the NCBI database, which is not only dedicated to plant genomes, EnsemblPlants is specifically dedicated to accessing plant genomes. EnsemblPlant is part of the Ensembl project that started in 1999, where the project aimed to automatically annotate the genome and integrate the outcome of the annotation with other publicly available biological data and establish an open access archive or database online for the use of the research community [11]. Ensembl project later launched the taxonomic specific websites designated for each taxon under their project that also includes the plants. The database is a user-friendly integrative platform, where it is continuously updated with the new addition of plant species every time a plant genome is completely sequenced. Compared to the NCBI database mentioned earlier, EnsemblPlant not only provides genome sequence, gene models, and functional annotation of the plant species of interest, but also includes the polymorphic loci, population structure, genotype, linkage, and phenotype information [11, 12]. Unlike, NCBI, EnsemblPlant does also provide comparative genomics data of the plant species of interest. This indicates that the platform does not only offer genome sequence data but provide additional analytical data about the plant species of interest and help the researchers who are working on plant bioinformatics to save a lot of time by reducing the tedious work in running the analysis. Yet, the researchers could re-assess the data if necessary, depending on the stringency of their work.

Aside from the abovementioned databases that are widely used for retrieving plant genome sequence, there are still other plant databases such as PlantGDB, MaizeDIG, and Phytozome that can also be considered. Table 1 lists the available database and tools that are widely applied in plant biotechnology.

**Table 1:** List of bioinformatics databases and tools applied in plant biotechnology

Function	Servers	URL	Details
Genome Database	ArrayExpress	www.ebi.ac.uk/arrayexpress	Archive for functional genomics data from microarray and sequencing platforms
	BarleyGenes	https://ics.hutton.ac.uk/barleyGenes/	Gene and RNA-seq database for Barley
	Chrysanthemum Transcriptome Database	http://www.icugi.org/chrysanthemum	Database of chrysanthemum’s gene and transcripts
	Cottongen	https://www.cottongen.org/	Genomics, genetics, and breeding database for cotton
	Expression Atlas EMBL-EBI	www.ebi.ac.uk/gxa/home	Open resource platform for gene and protein expression information
	Ensembl Plants	https://plants.ensembl.org/index.html	Genomic database (genome sequence, gene models, functional annotation) of more than 33 plant species
	Gene Expression Omnibus	www.ncbi.nlm.nih.gov/projects/geo	Functional genomics data repository
	MaizeDIG	https://maizedig.maizegdb.org/	Genotypic-phenotypic database for maize
	MaizeGDB	https://www.maizegdb.org/	Genetic and genomics database for maize
	MaizeMine	https://maizemine.rnet.missouri.edu/maizemine/begin.domine/begin.do	Archive to access literature, genomic, interaction and proteomic data for maize
	VvGDB	www.plantgdb.org/VvGDB/	Database for grape genome
	Phytozome	https://phytozome-next.jgi.doe.gov/	Comparative genomics portal of plants
	Plant Promoter Database	http://linux1.softberry.com/berry.phtml?topic=plantprom&group=data&subgroup=plantprom	Database of plant promoter sequences and experimentally determined transcription start site of various plant species
	PLEXdb	http://www.plantgdb.org/prj/PLEXdb/	Plant expression database with gene expression profile data sets, structural genomics, and phenotypic data
	Pomamo	https://www.gabipd.org/projects/Pomamo/	Archive of potato sequences, literature, maps, and tools
	PRGdb	http://prgdb.org/prgdb/	Archive of pathogen receptor genes of various plant species
	Rice Expression Database	http://expression.ic4r.org/	Repository of gene expression profiles of rice from RNA-seq data on tissues spanning an entire range of rice growth
	Rice expression profile Database	http://ricexpro.dna.affrc.go.jp/	Repository of gene expression profiles of rice from microarray analysis
	SolGenomics Network	https://solgenomics.net/gem/experimental_design.pl?id=2	Database and tool of genomics and genetics approach for tomato and some other plant species such as eggplant
	TAIR	https://www.arabidopsis.org/	Database of genetic and molecular biology data for *Arabidopsis thaliana*
	The Rice Annotation Project Database	https://rapdb.dna.affrc.go.jp/	Archive of rice genome sequence, structure, and function
	Tomato Functional Genomics Database	http://ted.bti.cornell.edu	Archive of tomato microarray data, metabolite and RNA-seq
Metabolomic Database	Metabolights	www.ebi.ac.uk/metabolights/	Metabolomics database (cross-species, metabolite structures, biological role)
Pathway Database	Reactome	www.reactome.org	Pathway interaction repository
	RiceCyc	http://pathway.gramene.org/gramene/ricecyc.shtml	Database of known and predicted biochemical pathways from rice
	PlantCyc	http://plantcyc.org	Metabolic pathway reference database from over 350 plant species
RNA Analysis Tool	qTeller	https://qteller.maizegdb.org/	Comparative RNA-seq expression tool
Chemical Compound Database	ChEBI	www.ebi.ac.uk/chebi/	Archive of molecular entities that focuses on small chemical compounds
	PubChem	http://pubchem.ncbi.nlm.nih.gov/	Archive of chemical information of various chemical compounds
Mass Spectrum Database	ReSpect for Phytochemicals	http://spectra.psc.riken.jp/menta.cgi/index	Database for phytochemicals MS spectra data and literature
	Metlin	http://metlin.scripps.edu/	Comprehensive MS/MS database
Resistance Analysis	Disease Resistance Analysis and Gene Orthology (DRAGO2)	http://prgdb.org/prgdb/drago2	Annotate resistance genes
Networking and Interaction Analysis	PathoPlant	www.pathoplant.de/	Database of plant-pathogen interaction and components related to plant pathogenesis
	AraNet	www.inetbio.org/aranet/	Probabilistic functional gene network tool for *Arabidopsis thaliana*
	PLANET	http://aranet.mpimp-golm.mpg.de/	Platform for visualization and analysis of co-function networks of photosynthetic organisms
Genomics Tool	The Bio-Analytic Resource	http://bar.utoronto.ca/	Plant bioinformatics tools resource platform (gene expression, mapping, molecular markers, and genomics)

### Biotechnology and bioinformatics for plant breeding

**Fig. 1:**
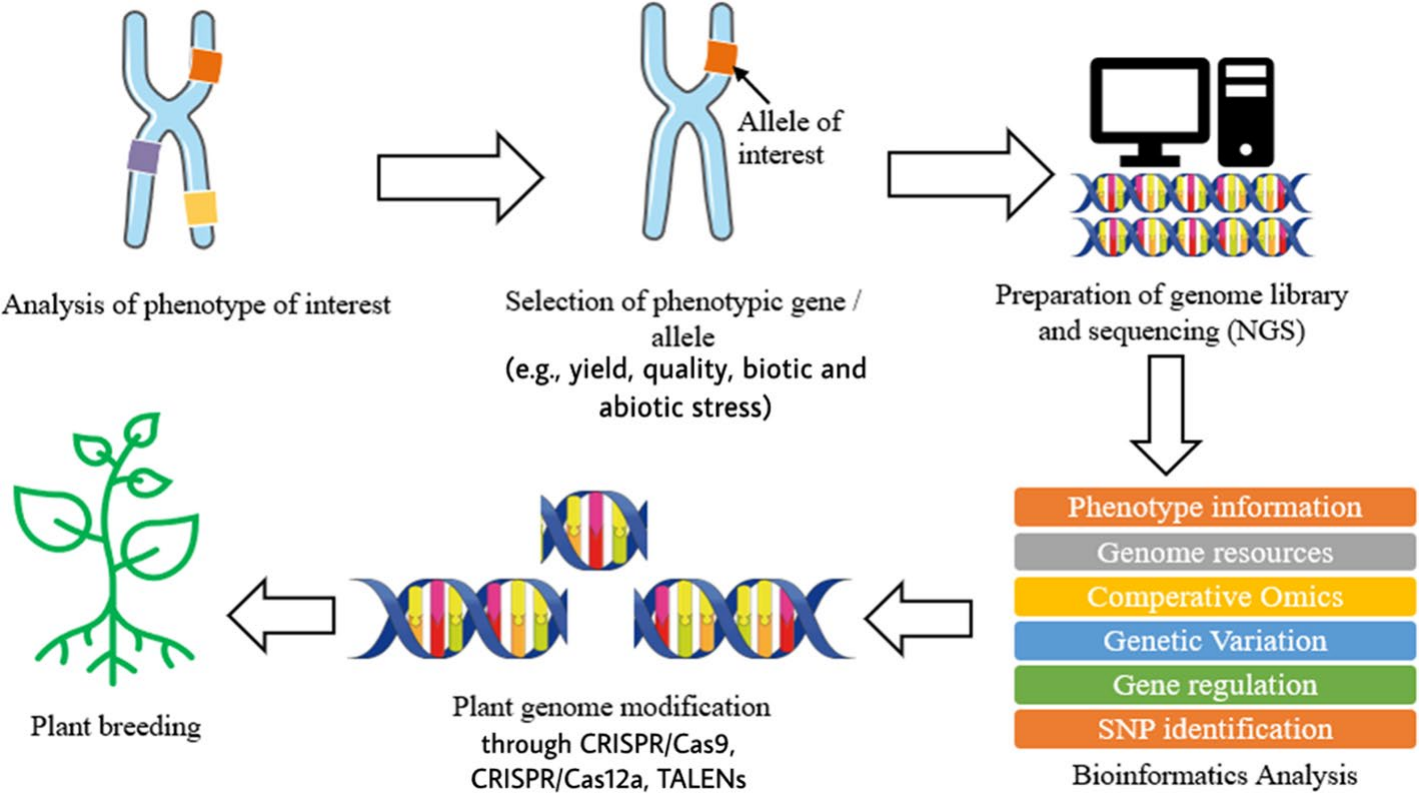
Brief process of plant breeding involving NGS and bioinformatics

Plant breeding can be defined as the changing or improvement of desired traits in plants to produce improved new crop cultivars for the benefits of humankind [8]. Jhansi and Usha [13] mentioned a few benefits brought by genetically engineered plants such as improved quality, enhanced nutritional value, and maximized yield. The revolution of life science in molecular biology and genomics has enabled the leaps forward in plant breeding by applying the knowledge and biological data obtained in genomics research on crops [6, 8, 13]. In modern agriculture, transgenic technology on plants refers to genetic modification, which is done on plants or crops by altering or introducing foreign genes into the plant, to make them useful and productive and enhance their characteristic [13, 14]. As mentioned above, the evolution of next-generation sequencing (NGS) and other sequencing technologies produces a large size of biological data which require databases to store the information. The accessibility of whole genome sequences in databases allows free association across genomes with respect to gene sequence, putative function, or genetic map position. With the aid of software, it is possible to formulate predictive hypothesis and incorporate the desired phenotypes from a complex combination into plants by looking at those genetic markers which score well and gives a higher reliability in breeding [2, 15]. Other than genome sequence information, databases which store the information of metabolites also play a crucial role in the study of interaction with proteomics and genomics to reflect the changes in phenotype and specific function of an organism [1]. Some of the most widely used metabolomics databases for plants and crops such as Metlin (http://metlin.scripps.edu), provides multiple metabolite searching and about 240,000 metabolites, nearly 72,000 high-resolution MS/MS spectra, and PlantCyc (https://plantcyc.org/), a database which stores information about biochemical pathway and their catalytic enzyme and genes from plants [1, 16]. Moreover, single-nucleotide polymorphism markers also benefit from the revolution of NGS and other sequencing technologies. By using NGS, RNA sequencing (RNA-seq) allows direct measure of mRNA profile in order to identify known single-nucleotide polymorphism (SNP) [1]. SNP is the unique allelic variation within a genome of same species, which can be used as biological markers to locate the genes associated with desired traits in plants [17, 18]. Besides, transcriptome resequencing using NGS allows rapid and inexpensive SNP discovery within a large, complex gene with highly repetitive regions of a genome such as wheat, maize, sugarcane, avocado, and black currant [17]. Figure 1 illustrates briefly the process involved in plant breeding using NGS and bioinformatics.

### Rice

Ever since the first transgenic rice production in 2000, there has been a significant revolution in crop genome sequencing projects, along with the advancement in technologies, rapidly increasing the pace in genetically modified organism (GMO) [2, 13, 19]. Among all the products in rice biotechnology, one of the most widely known GM rice is golden rice. Golden rice is a variety of rice engineered by introducing the biosynthetic pathway to produce β-carotene (pro-vitamin A) into staple food in order to resolve vitamin A deficiency. The World Health Organization has classified vitamin A deficiency as public health problem as it causes half a million of children to childhood blindness [13]. Vitamin A is an essential nutrient to humans as it helps with development of vision, growth, cellular differentiation, and proliferation of immune system; insufficient intake of vitamin A may lead to childhood blindness, anaemia, and reduced immune responsiveness against infection [20]. Being the first crop genome to be sequenced, rice has become the most suitable model to initiate the development and improvement of other species in genomic aspect [21–24]. The particular reason is due to its small genome size and diploidy, which enables rice to be an excellent model for other cereal crops with larger genomes, such as maize and wheat [21, 23]. Song et al. [22] reported the complete genome sequence of two rice subspecies, *japonica* and *indica*, in 2005 that laid a strong foundation for molecular studies and plant breeding research [22, 24]. With recent advancement in bioinformatics, it is now possible to run the sequence alignment between large and complex genome from other crop species with genomic data available from rice, by using different software or tools, in order to find out the shared conserved sequence through comparative genomics [2, 7]. Vassilev et al. stated some of the most commonly used programmes such as BLAST and FASTA format allowed rapid sequence searching in databases and give the best possible alignment to each sequence [25]. The programming algorithm calculates the alignment score to measure the proportion of homology matching residue between sequence from related species [2].

### Wheat

Wheat, as the most widely grown consumed crops, together with rice and maize contributes more than 60% of the calories and protein for our daily life [26, 27]. To meet the demands of human population growth, it is necessary to achieve more understanding in wheat research and breeding in order to accelerate the production of wheat yield by 2050 [26–28]. Despite its importance, the improvement of wheat has been challenging as the researchers have to overcome the complexity of the wheat genome such as highly repetitive and large polyploid in order to get a fully sequenced reference genome [26, 29]. Advances in next-generation sequencing (NGS) platforms and other bioinformatics tools have revealed the extensive structural rearrangements and complex gene content in wheat, which revolutionized wheat genomics with the improvement of wheat yield and its adaptation to diversed environments [26, 29]. The NGS platforms allow the swift detection of DNA markers from the huge genome data in a short period of time. These NGS-based approaches have undoubtedly revolutionized the allele discovery and genotype-by-sequencing (GBS). By providing a high-quality reference genome of wheat in databases, it allows more sequence comparison between wheat and other species to find out more homologous gene. Moreover, the development of sequencing technologies in both high-throughput genotyping and read length, combining with biological databases, allow the rapid development of novel algorithm to complex wheat genome [29, 30]. For instance, genome-wide association studies (GWAS) are an approach used in genome research which allows rapid screening of raw data to select specific regions with agronomic traits [29, 31]. It allows multiple genetic variants across genome to be tested to study the genotype-phenotype association; thus, this method can be used to facilitate improvement in crop breeding via genomic selection and genetic modification [16, 29].

### Maize

Maize, a globally important crop, not only has a wide variety of uses in terms of economic impact, but can also serve as genetic model species in genotype to phenotype relationship in plant genomic studies [32, 33]. Besides, due to its extremely high level of gene diversity, maize has high potential in the improvement of yield to meet the demands of population growth [33]. Despite the combination of economic and genomic impact, the progress in generating a whole genome sequence in maize has been a computational challenge due to the presence of tremendous structural variation (SV) in its genome [34]. The introduction of NGS techniques in several crops including maize allowed the rapid de novo genome sequencing and production of huge amount genomics and phenomics information [1, 35]. A better integration of data within multiple genome assemblies is much needed to study the connection between phenotype and genotype in order to achieve yield and quality improvement of maize [35]. Nowadays, some user-friendly online databases such as qTeller, MaizeDIG, and MaizeMine are designed to ease the comparison and visualization of relationships between genotypes and phenotypes [36]. MaizeGDB, a model organism database for maize, provides the access of data on genes, alleles, molecular markers, metabolic pathway information, phenotypic images with description, and more which are useful for maize research [35, 36]. MaizeMine is a data mining resource under MaizeGDB, which was designed to accelerate the genomics analysis by allowing the researchers to better script their own research data in downstream analysis [36] whereas MaizeDIG is a genotype-phenotype database which allows the users to link the association of genotype with phenotype expressed by image [35, 36]. Cho et al. [35] reported that with the accessibility via image search tool, the relationship between a gene and its phenotype features can be visualized within image. The integration and visualization of high-quality data with these tools enables quick prioritizing phenotype of interest in crops, which play a crucial role in the improvement of plant breeding.

### Bioinformatics for studying stress resistance in plants

The understanding of the stress response on plants is vital for the improvement of breeding efforts in agriculture, and to predict the fate of natural plants under abiotic change especially in the current era of continuous climate change [37]. Stress response in plants can be divided into biotic and abiotic. Biotic stress mainly refers to negative influence caused by living organism such as virus, fungi, bacteria, insects, nematodes, and weeds [38] while abiotic stress refers to factors such as extreme temperature, drought, flood, salinity, and radiation which dramatically affect the crop yield [37]. NGS technologies and other potent computational tools, which allowed sequencing of whole genome and transcriptome, have led to the extensive studies of plants towards stress response on a molecular basis [1, 2, 37]. The tremendous amount of plant genome data obtained from genome sequencing allows the investigation of correlations between the molecular backbone of living organism and their adaptations towards the environment [16].

#### Biotic and abiotic stress management

How the plants and crops respond towards stress environment is the key to ensure their growth and development, and to avoid the great crop yield penalty caused by harsh condition [35, 39]. Therefore, the utilization of bioinformatic tools is important to study and analyse the plant transcriptome in response to biotic and abiotic stress. Besides, the application of bioinformatics tools on plants and crops genome can benefit the agricultural community by searching the desired gene among genome from different species and elucidate their function on the crops [35]. The genome databases play a crucial role in storing and mining large and complex genome sequence from the plants. Besides data storage, some genome databases are also able to perform gene expression profiling to predict the pattern of gene expressed at the level of transcript in cell or tissues. By using in silico genomic technologies, the disease resistance gene-enzyme with their respective transcription factor, which plays a role in defence mechanism against stress, are able to be identified [40, 41]. For instance, a large-scale transcriptome sequencing of chrysanthemum plants was carried out by Xu et al. [40] to study the dehydration stress in chrysanthemum plants. An online database called Chrysanthemum Transcriptome Database (http://www.icugi.org/chrysanthemum) was developed to allow the storage and distribution of transcriptome sequence and its analysis result among research community [40]. With the aid of different protein databases, the biochemical pathway and kinase activity of chrysanthemum in response to dehydration stress are able to be predicted [40]. Xu et al. [40] also reported a total of 306 transcription factor and 228 protein kinase that are important upstream regulator in plants when encountered with various biotic and abiotic stresses.

### Bioinformatics approaches to study resistance to plant pathogen

One of the challenges in modern agriculture to supply the nutrition’s demand along with the world population growth is the crop loss due to disease. The study of plant pathogen plays an essential role in the study of plant diseases, including pathogen identification, disease aetiology, disease resistance, and economic impact, among others [41]. Plants protect themselves through a complex defence system against variety of pathogen, including insects, bacteria, fungi, and viruses. Plant-pathogen interaction is a multicomponent system mediated by the detection of pathogen-derived molecules in the form of protein, sugar, and polysaccharide, by pattern recognition receptor (PRRs) within the plants [42–45]. After the recognition of enemy molecules, signal transduction is carried out accordingly and plant immune systems will respond defensively through different pathways involving different genes [42]. According to Schneider et al. [46], the development of molecular plant pathology can be broadly divided into three eras, begins with the disease physiology starting from early 1900s until 1980s [46]. In the second era of molecular plant genetic studies, one or a few genes of bacterial pathogens were focused whereas the third era of plant genomic studies began in 2000 with the sequencing of genome, and the first complete genome of bacterial pathogen, *Xylella fastidiosa*, was obtained [46]. The recent advance in DNA sequence technologies allow researchers to study the immune system of plants on genomic and transcriptomics level [1, 41, 42]. Genomics has revealed the mystery and complexity and consequently the various information about phytopathogen. A clearer picture of plant-pathogen interactions in the context of transcriptomic and proteomics can be visualized through the application of different bioinformatics tools, which in turn made feasible the engineering resistance to microbial pathogen in plant [43].

#### PRGdb: bioinformatics web for plant pathogen resistance gene analysis

Plants have developed a wide range of defence mechanism against different pathogen and ultimately inhibit growth and spread of pathogen [47, 48]. Plant defence system is mediated by resistance (R) gene [47]. R gene plays an important role in defence mechanism. They encode for protein that recognizes specific avirulent (Avr) pathogen proteins and initiated the defence mechanism through one or more signal transduction pathway in a hypersensitive response (HR) [41, 47, 48]. However, the essential components needed for protein to exert their resistance are still unidentified [48]. With the intention to study and identify more novel R gene, high-throughput genomic experiments and plant genomic sequence are essential to explore their function and new R gene discovery [47]. In 2009, Plant Disease Resistance Gene database (PRGdb), a comprehensive bioinformatics resource across hundreds of plant species, was launched in order to facilitate the plant genome research on discovery and predict plant disease resistance gene [47, 48]. To date, PRGdb 3.0 has been released with 153 reference resistance genes and 177,072 annotated candidate pathogen receptor genes (PRGs) [49]. This database act as an important reference site and repository to all the research studies on exploration and use of plant resistance genes [48, 49].

**Fig. 2:**
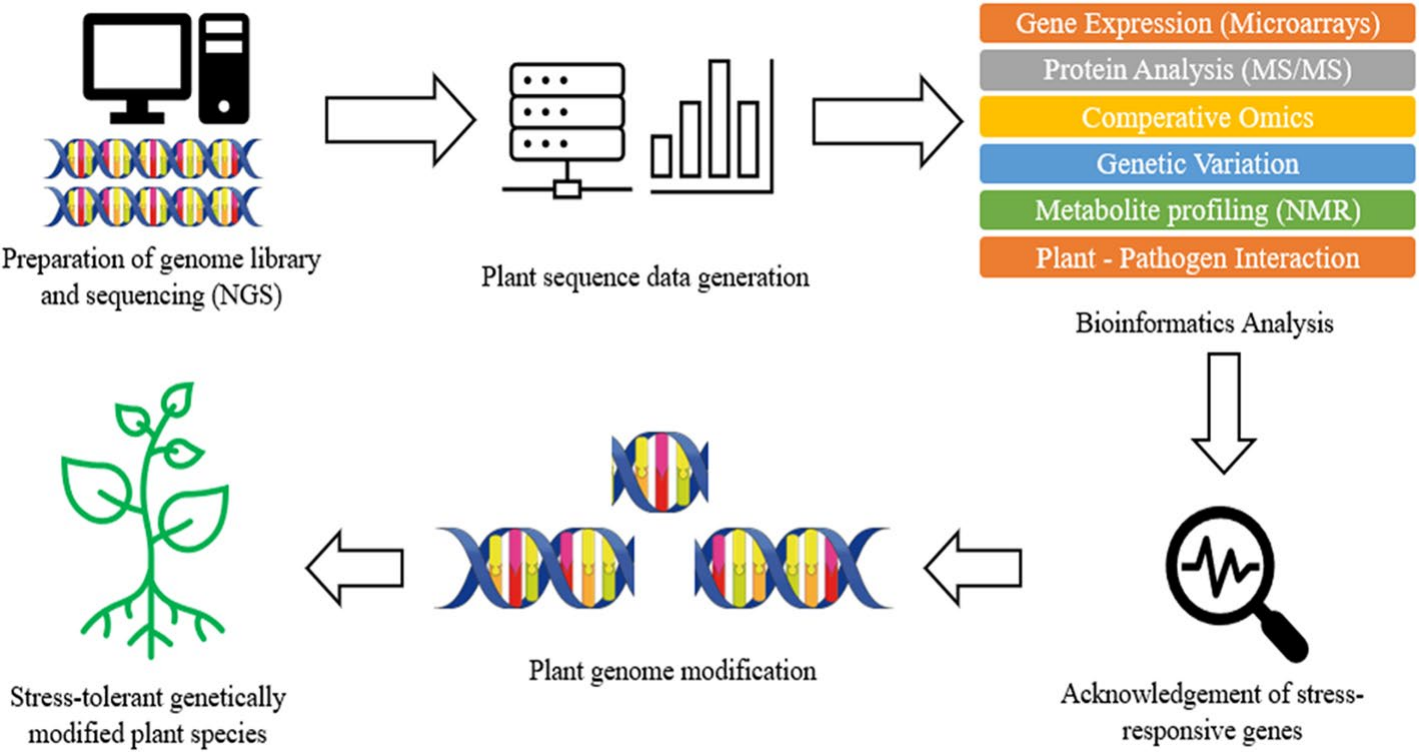
Brief process involved in producing stress-resistant plant using bioinformatics approach

Apart from resistance gene storage, this easily accessible platform also allows different tools that are essential for exploration and discovery of novel R gene. For instance, the DRAGO 2.0 tool, which was built to explore known and novel disease resistance gene, can be launched on any transcriptome or proteome to annotate and predict PRG from DNA or amino acid with high accuracy [49]. Besides, BLAST search tools available in PRGdb provide comparison of different sequences which allowed the determination of gene homology and expression analysis. Apart from the database, plant pathology field also benefited from whole genome sequence technologies. The new DNA sequencing technologies such as NGS and Sanger sequencing allowed the study of genomics, proteomics, metabolomics, and transcriptomics on both the host plant and the pathogen [1]. The phytopathogen genomes which have been sequenced are expected to provide valuable information on the molecular basis for infection of plant host and explore the potential novel virulence factors [1]. Figure 2 illustrates a brief process involved in producing stress-resistant plant using bioinformatics approach.

### Metagenomics in plant biotechnology and Cas9 modification

The effects of environment microorganisms’ community, especially soil microorganism on plants, may contribute to plant’s growth and pathogenesis. Through metagenomics approaches, the soil microorganism community that contributed to plant growth may provide a great genomic insight into physiology and pathology [50–53]. In metagenomics approaches, the overall genetic materials obtained from soil are sequenced and advancing to microbial community analysis via data analytics [53–55]. The extracted genetic materials from the soil were subjected to high-throughput metagenomics analysis via various NGS approaches such as 16S rRNA sequencing, shotgun metagenomic sequencing, MiSeq sequencing [54–56] for microbial species identification, functional genomics study, and structural metagenomic analysis. A NGS produces huge genomics data for each study; thus, application of bioinformatics tools would add value in the metagenomics analysis as the target genes identified could advance into elucidation of plant growth, plant disease, soil contamination, and microbial taxonomy [52]. For example, the use of UNITE (https://unite.ut.ee/) for fungi identification [57], SILVA (https://www.arb-silva.de/) for 16S rRNA [58], and MGnify (https://www.ebi.ac.uk/metagenomics/) possesses metagenomics data of microbiome [59]. These databases allow the researchers to retrieve and analyse the relevant metagenomic sequenced data for a specific study.

Since metagenomics analysis provides the greater output on plant-microbe interaction, the genes that are responsible for plant immunity may play a crucial role in protecting against disease-causing microorganism [60, 61]. With the emergence of Clustered Regularly Interspaced Short Palindrome Repeats (CRISPR) gene editing technique, Cas9 modification could produce a better plant trait and disease-resistant plant [62, 63]. The CRISPR/Cas9 system is employed in studying the functional genomics in plants in relation to plant-microbe interaction. CRISPR/Cas9 system facilitated the gene editing by creating a mutant through double-stranded break forming a targeted gene mutation and followed by genome repair [63–65]. The CRISPR/Cas9 modification on *OsSWEET14* genes protects the Super Basmati Rice from bacterial blight causes by *Xanthomonas oryzae pv. oryzae* [66]. Gene editing to knockout *OsMPK5* and *OsERF922* genes in rice protects against *Magnaporthe grisea* and *Magnaporthe oryzae*, respectively [67–69]. Besides that, Cas9 modification on Cs*WRKY22* and *TcNPR3* increased host defence immunity through regulating salicylic acid in *Citrus sinensis* and *Theobroma cacao*, respectively [70, 71]. Thus, CRISPR/Cas9 modification could be one of important science advancements to validate the metagenomics analysis on plant-microbe interaction.

#### Current challenges of bioinformatics applications in plant biotechnology

Despite the beneficial prospect of the bioinformatics applied in plant biotechnology, there are many challenges and limitations must be addressed in order to fully utilize their potentials [1]. Along with the rapid growth in plant genome data mining and database development, there are a few challenges faced by bioinformaticians and scientists which can be divided into number of areas as mentioned in the subsections below.

### Bioinformatic data management and organization and synchronize update resources

Since the introduction of the next-generation sequencing (NGS), which is commercially available in 2004, enormous amount of data has been generated in plant genome research. Thousands of Gb of plants sequences are deposited in various public databases monthly [1, 72, 73]. Moreover, the constantly sequenced and re-sequenced of the plant genome has developed a vast amount of new genome sequence in all public databases. The increase in sequenced plant genome driven by technological improvement has led to a problem that arises along with the storage and update of a large amount of data [72, 74]. The update process should occur in all the comparative databases, not just solely individual genome database [72]. With this, the synchronized update of genome data resources among different plant genomic platform is able to provide a strong, updated, reliable database community that all the plant researchers can rely on [72].

### Complexity of plant genetic content

Other than the tremendous amount of genome sequence generated, the complexity of the plant genetic content is also a challenging issue faced by plant research community. Even though the arrival of next-generation sequencing technologies has allowed the rapid DNA sequencing for non-model or orphan plant species, the sequencing pace for plants is far from that of animal and microorganism [74]. The main factor which contributes to this situation is because sometimes the plant genome can be nearly hundred times larger than the currently sequenced animal and microorganism genome [73]. Needless to say, some of the plant genome even can have polyploidy, a duplication of an entire genome, which is estimated to occur in 80% of the plant species [73, 75]. According to Schatz et al., the genome assembly in the case of large size plant genome with abundance of repetitive sequence can be metaphorically described as build-up of a large puzzle consisting of blue sky separated by nearly indistinguishable white clouds of small gene [73]. The particular reason for this is mainly because the sequence length in NGS is relatively shorter than in Sanger sequencing and required dedicated assembly algorithm [74]. Therefore, most plant genomes sequenced by NGS can only be used for establishing gene catalogues, interpreting the repeat content, glimpsing evolutionary mechanism, and performing on comparative genomics in early study [74].

### Advance in sequencing technologies

There are two basic approaches to genome assembly, i.e. comparative genome assembly and de novo genome assembly [75]. It is important to distinguish between these two different approaches. Comparative is a reference-guided method which use a genome or transcriptome, or both, for guidance, whereas de novo assembly refers to reconstruction of a genome from organisms that have not been sequenced before [74, 75]. Table 2 compares some of the available assembly and NGS technology available for genome sequencing. However, these two approaches are not completely exclusive due to a lack of bioinformatic tools designed to cope with the unique and challenging features of plant genomes [74, 75]. One of the biggest challenges in the development of bioinformatic software is the algorithm development [76]. As is known, all the programmes or software in bioinformatic are very computationally intensive. As most of the assemblies available now solely rely on single assembly, a development in better algorithm in terms of resource requirement is essential for combining different assemblers by using a different underlying algorithm in order to give a more credible final assembly [74, 76].

**Table 2:** Comparison between next-generation sequencing technologies

Method	Illumina	Pacific Bio	Nanopore	Pyrosequencing (454)	SOLiD
Read length per run	50–300 base pair	10–25 kilo base pair	500–2.3 mega base pair	Approximate 800 base pair	50 base pair
Time taken per run	1 to 10 days	Up to 30 h	1 min–72 h	24 h	1 to 2 weeks
Cost	$148 per Gb	$2000 Gb	$60–80 per sample	$7000 per sample	$15,000 per 100 Gb
Accuracy	98%	99.9%	98.9–99.6%	99.9%	99.9%
Advantages	Cost-effective, high-yield sequence reads	Fast, long read lengths	Real-time analysis, long read lengths	Fast, long read lengths	High accuracy
Disadvantages	Instrument cost, high maintenance of instrument, read length	Low high throughput	Error prone	Homopolymer error	Long run time, low read length

### Database accessibility

To date, there are about 374,000 known plant species in the world [77]. The first full plant genome sequencing was completed on A*rabidopsis thaliana* through Sanger sequencing methods in 2000 [78]. Although introduction of molecular biology decades ago may have facilitated the species identification, obtaining the full plant genomic data remains challenging due to the genome complexity. The development of NGS platform may foster the plant genome sequencing, yet there are limited sequenced datasets reposited to the database. To date, there are only 29 plant genome databases accessible in PlantGDB genome browser allowing researchers to retrieve the information about gene structure, matched GSS contigs, similar protein, spliced alignments EST, etc. Besides, the PlaD database (http://systbio.cau.edu.cn/plad/index.php) that focuses on the microarray data of the plants developed by China Agricultural University comprises transcriptomic database for plant defence against pathogen. However, it is limited to *Arabidopsis*, rice, maize, and wheat [79]. The Plant Omics Data Center (http://plantomics.mind.meiji.ac.jp/podc/) is another publicly available web-based plant database featuring omics data for co-expressed profile, regulatory network, and plant ontology information [80]. Although curated omics datasets could be retrieved from PODC, information are restricted for certain plants and crops such as *Arabidopsis*, tobacco, earthmoss, barrelclover, soybean, potato, rice, tomato, grape, maize, and sorghum. Furthermore, all these publicly available databases require constant updating with new released data or resequencing data so that the researcher could obtain the most updated version of genome datasets for their research.

## Conclusion

The application of bioinformatics in plant biotechnology represents a fundamental shift in the way scientists study living organisms. Bioinformatics play a significant role in the development of agriculture sector as it helps to study the stress resistance and plant pathogen, which are critical in advancing crop breeding [75]. NGS and other sequencing technologies will make more plant genome data accessible in all public databases and enable the identification of genomic variants and prediction of protein structure and function [75, 76]. Moreover, GWAS, which allows the identification of loci and allelic variation related to valuable traits, eased the crop modification and improvement [74]. In brief, the advance in bioinformatics application in plant biotechnology enables researchers to achieve fundamental and systematic understanding of economically important plant. However, despite all these exciting achievement by the application of bioinformatic on plant biotechnology, it is still a long way from automated full genome sequencing and assembly at a low cost [76]. There is a critical need for effective bioinformatic tools which are able to provide longer reads with unbiased coverage in order to overcome the complexity of the plant’s genome. To achieve this, an enhanced algorithm development is essential to enable data mining and analysis, comparison, and so on. Therefore, bioinformaticians and experts with mathematical and programming skills will play an important role in bringing fresh approaches and knowledge into bioinformatics, not only for the advancement in plant biotechnology and agriculture sector, but the future of humanity as well.

## Data Availability

Not applicable.

## Abbreviations

GWAS: Genome-wide association studies
NGS: Next-generation sequencing
PRGdb: Plant Disease Resistance Gene database
RNA-seq: RNA sequencing
SNP: Single-nucleotide polymorphism
